# Stercoral Perforation Requiring Subtotal Colectomy in a Patient on Methadone Maintenance Therapy

**DOI:** 10.1155/2012/176143

**Published:** 2012-06-17

**Authors:** A. Sakharpe, Y. K. Lee, G. Park, V. Dy

**Affiliations:** ^1^Department of Surgery, Easton Hospital, Easton, PA 18042, USA; ^2^Drexel University College of Medicine, Philadelphia, PA 19129, USA

## Abstract

Stercoral perforation of the colon is a rare but serious complication of chronic constipation. We present a case of stercoral perforation requiring subtotal colectomy in a 41-year-old female who had been on methadone maintenance for a history of long-term intravenous heroin use. Our case highlights the importance of prompt and thorough surgical intervention in the successful treatment of this rare condition.

## 1. Introduction 

Stercoral perforation is recognized as a rare cause of colonic perforation that is most commonly seen in elderly, debilitated, and/or institutionalized patients who are immobile and on multiple medications. Many drugs such as narcotics, anticholinergics, antacids, and NSAIDs have been implicated as causes of fecal impaction resulting in stercoral perforation. The mortality has been reported to be as high as 57%, but prompt surgical exploration may decrease that rate [1]. 

We report a case of subtotal colectomy performed for stercoral perforation in an otherwise healthy individual who was on methadone maintenance for a long history of intravenous heroin use. 

## 2. Case Presentation 

A 41-year-old Caucasian female presented with a 2-day history of abdominal distention and generalized pain that was worsened with motion. These symptoms were accompanied by nausea, several episodes of nonbilious vomiting, and poor appetite. Her last bowel movement had been a week prior to admission. Her past medical history was significant for type 2 diabetes and vulvar lichen sclerosis, which was being treated with steroids since its diagnosis one year prior. She had a 15-pack-year history of tobacco use, as well as a distant history of intravenous heroin abuse with subsequent long-term dependence on methadone maintenance at 180 mg. 

**Figure 1:**
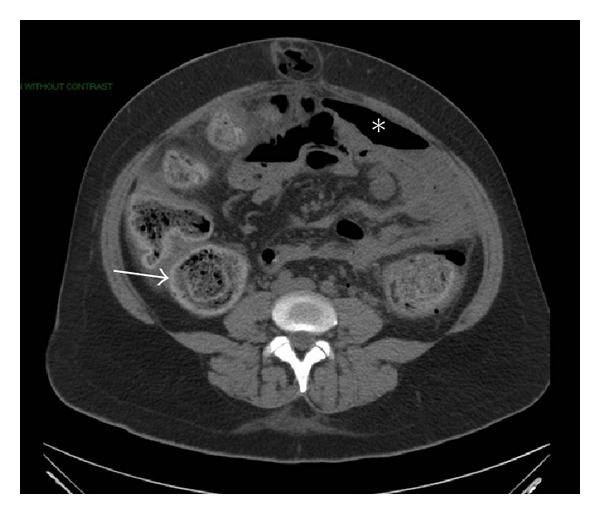
CT scan of the abdomen with free air (star) and colonic wall thickening (arrow).

**Figure 2:**
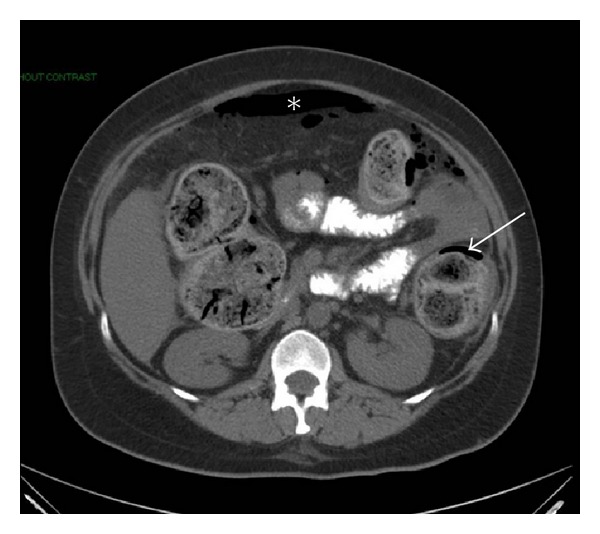
CT scan of the abdomen with free air (star) and air in bowel wall (arrow).

Vital signs were noted as temperature 98.7°F, blood pressure 121/68 mmHg, pulse 101 bpm, and respiratory rate 24. Physical examination revealed diffuse abdominal tenderness with maximal pain on the left side, as well as rebound tenderness, guarding, and hypoactive bowel sounds. A rectal exam revealed no masses, empty ampulla, and hemoccult negative stool. Laboratory studies revealed a white blood cell count of 22,200/mL with bandemia, glucose at 267 mg/dL, and lactic acid elevated at 4.7 mmol/L. Other laboratory values were all normal. A CT scan of the abdomen and pelvis with PO contrast revealed multiple foci of extraluminal air in the peritoneum, a large amount of fecal material in the colon, and extravasation of oral contrast consistent with perforation of the colon (Figures 1 and 2).

**Figure 3:**
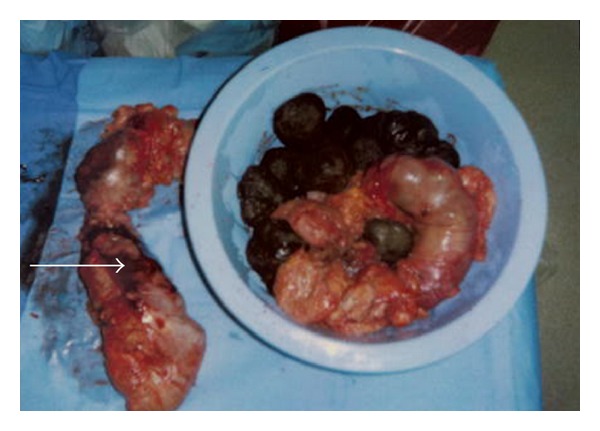
Resected specimen with multiple large fecaliths of varying size and serosal hemorrhage (arrow) on bowel wall.

The patient was promptly taken to the operating room for an exploratory laparotomy with a provisional diagnosis of bowel perforation with peritonitis. She was found to have a large perforation in the sigmoid colon measuring approximately 8–10 cm. The remainder of the bowel was proximally dilated and filled with hard dry fecal material, with its widest point measuring 19 cm in circumference with a very thin bowel wall. Extensive purulent material with fibrinous exudates into the small and large bowel, as well as multiple large fecaliths of varying size in the peritoneal cavity, was found. Initially, an attempt was made to salvage part of the colon by pursuing a left hemicolectomy, but further exploration revealed extensive serosal hemorrhage of the transverse colon, which prompted the decision to pursue a subtotal colectomy with end ileostomy (Figure 3). 

Pathological examination of the resected colon revealed multiple ischemic mucosal ulcers consistent with sterocoral perforation. The margins of the perforation showed transmural necrosis with acute necroinflammatory changes. No other pathologies like infarction, inflammatory bowel disease, or tumors were found so fecalomas were attributed as the cause of serosal hemorrhage and colonic inflammation. 

The postoperative course was complicated by sepsis, mechanical ventilation dependence, and adult respiratory distress syndrome. She made a slow but satisfactory recovery and was subsequently discharged from the hospital 3 weeks afterwards. She returned 7 months later for reversal of ileostomy with an ileorectal anastomosis. 

## 3. Discussion

Stercoral perforation has been defined as perforation of the large bowel due to pressure necrosis from fecal masses [1]. It is recognized as a rare cause of colonic perforation, with only 81 cases reported in the literature since its first description in 1894 [2, 3]. However, a prospective study by Maurer et al. found stercoral perforation to account for 1.2% of all emergency colorectal surgeries and 3.2% of all colonic perforations, suggesting that the true incidence may be higher than previously thought [2]. 

The mean age of presentation is 59 years with an equal gender distribution [2]. Most patients report a history of chronic constipation, as well as straining bowels as a precipitant event. There have been case reports of stercoral perforation in association with antipsychotics, tricyclic antidepressants, verapamil, aluminum-based antacids, immunosuppressive agents after renal transplants, nonsteroid anti-inflammatory drugs (NSAIDs), and opioids including codeine, heroin, and methadone [4–6]. These drugs are thought to affect colonic motility rather than the colonic wall itself.

Patients typically present with an acute onset of generalized abdominal pain, history of constipation, diffuse peritonitis, and free air on plain film. Early surgical intervention is critical to a good outcome, with the procedure of choice with the lowest mortality (23%) being resection of the diseased segment of colon, end colostomy, and Hartmann's closure of the rectum [7]. Moreover, even if stercoral ulcers are not visible to the surgeon, it is recommended that any substantially dilated colon, especially in the presence of multiple fecalomas, be removed up to subtotal colectomy in order to prevent a second perforation at a later time [2]. In the case of our patient, subtotal colectomy was pursued upon discovery of significant extensive serosal hemorrhage of the remaining colon along with multiple large fecaliths of varying size all along the colon.
